# Multi-Omics and Digital Biomarkers for Dynamic Cardiometabolic Risk Prediction

**DOI:** 10.3390/biomedicines14102263

**Published:** 2026-10-06

**Authors:** Kexin Ding, Xiaoyi Li, Jun Zhang, Dafang Chen

**Affiliations:** 1 Department of Epidemiology and Biostatistics, School of Public Health, Peking University, Beijing 100191, China; 2 Key Laboratory of Epidemiology of Major Diseases ( Peking University), Ministry of Education, Beijing 100191, China; 3 Hainan General Hospital, Affiliated Hainan Hospital of Hainan Medical University, Haikou 570311, China

**Keywords:** cardiometabolic disease, multi-omics, digital biomarkers, longitudinal monitoring, dynamic risk prediction, personalized reference intervals, wearable sensors, precision medicine

## Abstract

Cardiometabolic risk changes as metabolic state, vascular injury, behavior and treatment evolve. Multi-omics assays characterize the underlying molecular state, whereas wearable sensors and continuous glucose monitoring capture physiological responses during daily life. This review asks whether combining these measurements improves a defined clinical decision beyond established clinical information in type 2 diabetes, atherosclerotic cardiovascular disease and heart failure. We distinguish biological association, prediction and clinical utility, and define dynamic risk prediction as repeated estimation of future event probability over a specified horizon using only information available at each prediction time. Large cohorts link proteins and metabolites with future disease, and intensive studies connect molecular variation with activity, nutrition and glycemic responses. However, evidence for joint molecular–digital integration remains predominantly observational. Trials of glucose monitoring and structured telemanagement show that monitoring can improve outcomes when it is embedded in an effective care pathway, but they do not establish the incremental value of multi-omics. We therefore organize the evidence around three requirements: temporal alignment of stable genomic information, intermittent molecular assays and dense digital trajectories; validation against a strong clinical comparator; and prospective assessment of patient outcomes, cost and burden. Emerging sensors, dynamical network biomarkers and digital twins may eventually extend this framework, but they remain experimental or conceptual for integrated cardiometabolic event prediction.

## 1. Introduction

Cardiometabolic risk is usually assessed at isolated visits, although many of its determinants change between visits. Blood pressure, lipid concentrations and glycemic status measured in the clinic remain clinically informative, but they do not show how meals, sleep, activity, illness and treatment shape physiology from day to day. The gap matters differently across the disease course. In primary prevention, the task is to estimate long-term risk before a first event. In established heart failure (HF), the more immediate task may be to recognize deterioration over days. The cardiovascular–kidney–metabolic framework captures the biological links across these stages and explains why organ function must be considered alongside conventional risk factors [1].

Established risk equations remain the foundation of prevention. SCORE2 estimates cardiovascular risk in European populations, whereas PREVENT incorporates cardiovascular, kidney and metabolic information to estimate risks that include atherosclerotic cardiovascular disease (ASCVD) and HF [2,3]. These models turn population evidence into individual absolute-risk estimates. Any new molecular or digital marker should therefore be judged against the information already used in practice, not against an artificially weak model. The useful question is specific: for which population, outcome, prediction horizon and clinical action does the additional measurement improve a decision?

Two developments make that question timely. High-throughput assays now measure thousands of circulating proteins and metabolites at population scale, bringing molecular pathways into epidemiological studies of disease [4]. Digital devices repeatedly measure activity, heart rate, rhythm and glucose outside the clinic, supplying temporal and behavioral context that a blood sample cannot provide. Previous reviews have established the systems-biology rationale for multi-omics in cardiovascular disease, connected molecular discovery with wearable sensing, and described practical challenges in data integration [5,6,7,8,9]. The present review takes the next step: it asks what molecular and digital measurements contribute jointly, and what evidence would be needed before their integration could guide care.

Molecular and digital measurements address different clinical targets and timescales. A protein measured once may forecast an event years later, while a week of continuous glucose monitoring (CGM) can characterize meal responses and a wearable patch can detect changes before HF hospitalization. Baseline risk assessment estimates future event probability using information from one index assessment. Dynamic risk prediction updates that probability as a person remains at risk and new information becomes available, for a specified future interval [10]. Monitoring describes repeated observation of a health state or signal and need not produce an event probability. Early detection seeks to recognize disease or deterioration before usual clinical recognition. It overlaps with dynamic prediction when a system repeatedly estimates future event risk, but an abnormality flag or warning interval alone does not establish a calibrated risk estimate. The distinction concerns the target and output, rather than simply the length of follow-up.

**Figure 1:**
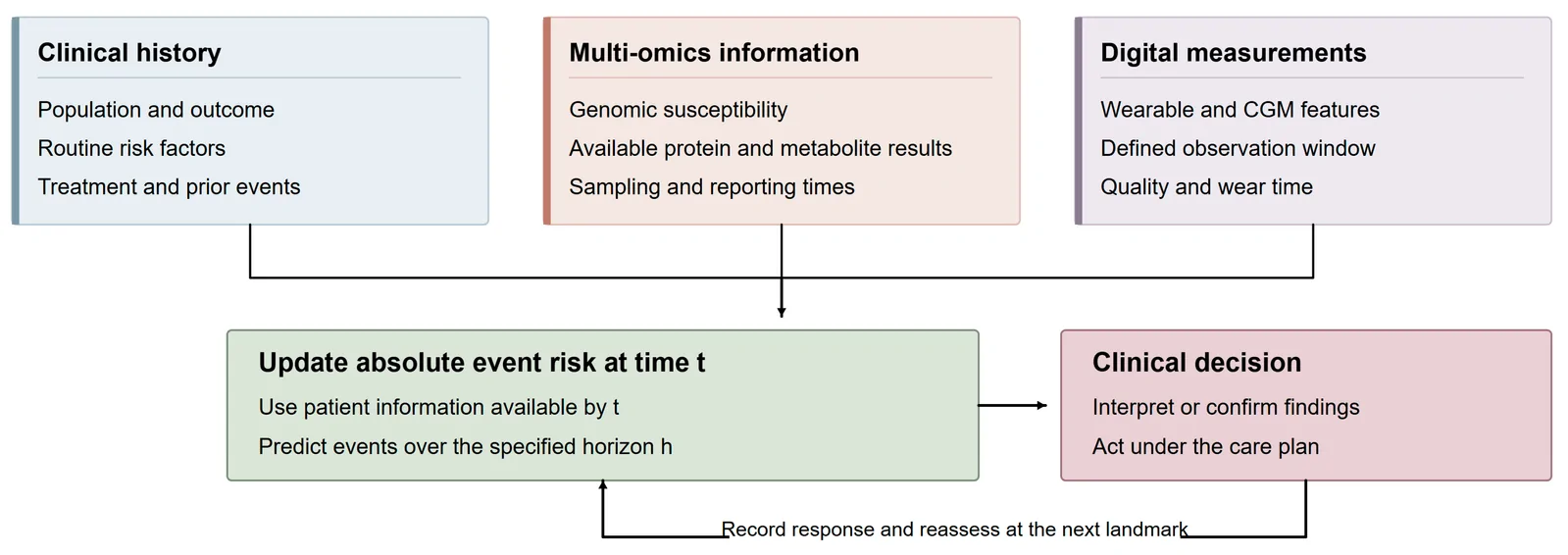
Proposed pathway for dynamic molecular–digital risk assessment. Clinical, molecular and digital information is combined to update absolute risk and support a defined clinical decision, followed by reassessment as new information becomes available.

Recent proposals for personalized cardiometabolic monitoring provide a conceptual basis for combining these tasks [11]. A related digital-intelligent precision-health framework connects multidimensional phenotyping with risk assessment and decision support [12]. Building on these proposals, Figure 1 specifies a narrower sequence: measurements available at a clinical decision point inform an updated absolute-risk estimate, that estimate supports a defined action, and subsequent observations permit reassessment. The sequence is testable, but its clinical value cannot be inferred from association studies alone.

This narrative review focuses on adults at risk of, or living with, type 2 diabetes (T2D), ASCVD and HF. We identified literature through targeted PubMed and publisher searches and reference tracing from June to August of 2026, followed by focused methodological and terminology searches during revision in September 2026. Recent human studies, particularly those published in 2024–2026, were prioritized, while foundational studies and methodological guidance were retained when they were needed for interpretation. The review first explains why molecular and digital measurements may be complementary, then examines what human studies have actually established, and finally sets out the modeling, validation and implementation steps needed to turn repeated measurements into clinical decisions.

## 2. What Molecular and Digital Measurements Contribute

### 2.1. Susceptibility, Molecular State and Physiological Response

Molecular layers differ in the information they carry and in how quickly that information changes. Germline variation is relatively stable and mainly describes inherited susceptibility. Transcripts, proteins and metabolites are more responsive to age, organ function, medication and environmental exposures, and therefore provide a changing view of molecular state. Genetic and molecular data can also clarify one another. For example, a genetic association with a circulating protein may reflect altered expression, processing or binding rather than a simple change in protein production [4,13].

Among molecular measurements, circulating proteins and metabolites are particularly accessible for clinical studies. They reflect processes relevant to T2D, ASCVD and HF, including substrate handling, insulin signaling, inflammation, endothelial function, myocardial stress and tissue remodeling. Their interpretation nevertheless depends on the source of the measured concentration. In the Fenland study, 4775 protein targets were profiled in 8350 participants, and plasma abundance varied with genetic, lifestyle, clinical and technical factors [14]. This large-scale analysis provides empirical support for interpreting molecular signatures in the context of the processes and exposures that shape their measured concentrations, rather than treating such factors solely as covariates to be adjusted away. Accordingly, factors such as kidney function, adiposity, and treatment history may be relevant to the biological interpretation of a molecular signature.

Digital measurements add a different kind of information. Activity, rest-activity rhythms, heart rate, rhythm and CGM record how physiology unfolds during daily life. Their value often lies in the trajectory and its context rather than in an isolated value. Reduced activity accompanied by a sustained rise in resting heart rate, for example, has a different interpretation from reduced activity during planned rest. Likewise, a glucose excursion is easier to interpret when meal timing, composition and preceding activity are known [15]. Molecular assays can therefore characterize biological state, while digital measurements show when and under what conditions that state is expressed.

This review uses multi-omics for analysis of at least two molecular layers and multimodal integration for combinations that may also include clinical or digital data. We use digital measurement, signal or feature for sensor-derived data unless the measure has been validated as a biomarker for a specified biological purpose. This follows the FDA–NIH BEST definition of a biomarker and the distinction between technical verification, analytical validation and clinical validation [16,17]. The terminology keeps the focus on what each measurement contributes to the clinical task. Table 1 summarizes those contributions and the main considerations for longitudinal use.

### 2.2. Connecting Metabolic and Vascular Pathways

Molecular and digital measurements become complementary when they reduce different uncertainties. A circulating signature may reveal altered substrate metabolism before diagnostic glucose thresholds are crossed, as shown by the association of branched-chain and aromatic amino acids with future diabetes in initially normoglycemic individuals [22]. However, a single concentration cannot show whether the finding is persistent or reflects a recent meal, tissue use or organ clearance. Repeated digital observations or a standardized challenge can supply that missing temporal context by showing whether an abnormal molecular state is accompanied by a reproducible physiological response.

Inflammation provides an example of a process shared across metabolic and vascular disease. Experimental studies link ceramide-related metabolic stress and cholesterol crystals to NLRP3 inflammasome activation and interleukin-1β release [23,24]. CANTOS provided clinical evidence that this pathway is relevant to recurrent cardiovascular events. Among 10,061 patients with previous myocardial infarction and high-sensitivity C-reactive protein of at least 2 mg/L, canakinumab at 150 mg every three months reduced the primary composite outcome without lowering lipid levels (hazard ratio 0.85, 95% confidence interval 0.74–0.98), although fatal infection was more frequent [25]. This evidence supports inflammatory proteins as biologically relevant molecular signals. Digital changes such as reduced activity or altered heart rate could help indicate when those signals occur during deterioration or acute illness, but the added predictive value of combining them must be tested directly. Intercurrent infection is one reason to interpret concurrent molecular and physiological changes cautiously, because it can alter both inflammatory profiles and metabolic control [26].

Epigenetic regulation may connect inflammatory signaling, mitochondrial function and cardiac or vascular remodeling, but effects differ by enzyme subtype and tissue context, and cardiovascular therapeutic evidence remains largely preclinical [27]. Transcriptional and epigenetic measurements may therefore help explain a circulating protein profile, yet explanatory value should not be confused with predictive value. The latter must be shown in clinical cohorts against simpler measurements.

The common lesson is not that more modalities are automatically better. Molecular panels can identify metabolic dysfunction, inflammation or organ stress, whereas digital observations can show when these patterns are expressed and whether they coincide with meals, activity, illness or functional decline. Integration is useful when it resolves an uncertainty left by either modality alone, such as whether a signal is persistent, exposure-related or part of an acute illness.

### 2.3. Biological Timing and Individual Variation

Temporal alignment is central to molecular–digital integration because the modalities change at different rates. Genomic susceptibility is largely fixed, many circulating molecules change over hours to months, and digital signals can vary from minute to minute. Measurements should therefore be repeated only often enough to capture the change relevant to the clinical question. Combining a months-old molecular profile with a current wearable trajectory may describe the same person without describing the same biological state.

Longitudinal multi-omics studies show why timing and context matter. Serial transcriptomic, proteomic and metabolomic profiles in one individual captured departures from a personal baseline across changing health states [28]. Studies in 106 and 109 participants extended repeated molecular and clinical profiling to cohort settings and showed how illness, weight change and other perturbations can shift an individual’s profile [19,29]. In 36 participants studied before and after acute exercise, 17,662 analytes followed different recovery trajectories, with altered responses among participants with insulin resistance [30]. A changing molecular value can therefore reflect an expected response to a stimulus rather than disease progression.

Personal baselines and population-reference ranges provide complementary information. A population range indicates how a person compares with others, while a within-person trajectory shows whether that individual has changed. Both are needed because a person may remain at high absolute risk despite a stable recent trajectory, or may show a clinically important deviation while remaining within the population range. The reference state may also need to be re-established after treatment, illness or a major behavioral change.

Sampling schedules should follow these biological timescales and the intended decision. Genotyping rarely needs repetition. A targeted protein or metabolite panel may be most informative around a clinical transition or defined challenge, while digital signals can be summarized daily or around relevant events. Additional sampling is useful only if it separates meaningful change from ordinary variation. Otherwise, it adds correlated observations, technical variation and participant burden without improving the decision. Selecting appropriate intervals remains an important research question [31].

### 2.4. Measurement Quality and Emerging Molecular Access

Reliable integration begins with measurement quality. Molecular assays differ in coverage, analytical sensitivity and measurement scale. Eldjarn and colleagues compared Olink results from more than 50,000 UK Biobank participants with SomaScan data from approximately 36,000 Icelandic participants. A subset of 1514 Icelandic samples had measurements on both platforms [13]. Correlations between paired measurements were modest overall, and some genetic associations differed between platforms. The association datasets and paired comparison address complementary aspects of assay performance and indicate a need to reassess signatures when transferring between technologies. Sample handling, fasting status, collection time and batch allocation can also alter apparent differences. Longitudinal studies should use quality-control specimens spanning the study period to distinguish laboratory batch effects from biological change.

Digital measurements have corresponding dependencies. Sensor position, wear time, motion and firmware affect the features available to an algorithm. Photoplethysmographic and electrocardiographic estimates of beat-to-beat variation require modality-specific validation. CGM measures interstitial glucose, and management targets should be applied in the populations and settings for which they were developed [21]. The V3 framework separates verification of technical performance, analytical validation against an appropriate reference and clinical validation for the intended use [17]. V3+ adds usability validation to assess whether intended users can operate the technology in the relevant context [20]. Together, these components address measurement quality, clinical relevance and practical use.

New sampling and sensing approaches may reduce the mismatch between molecular and digital timescales. Shen and colleagues developed multi-omics profiling from 10-µL blood microsamples [32]. One application characterized responses to a standardized nutritional shake in 28 participants at five time points. A separate proof-of-concept application paired frequent microsampling with wearable measurements in one individual over seven days. The wearable experiment demonstrated the feasibility of dense molecular–physiological observation at an individual scale. Reviews by Sempionatto and colleagues and Kim and colleagues describe how wearable chemical sensing could translate selected analytes from discovery studies into repeated monitoring [6,7]. Sweat, skin-associated fluids and interstitial fluid differ from plasma in transport and local production, so each sensed signal needs validation against its intended biological and clinical target.

Other circulating materials may add tissue information. Cardiomyocyte-specific DNA-methylation patterns have enabled detection of cardiac cell death in circulating cell-free DNA [33]. Extracellular vesicles provide another source of molecular cargo, although source attribution, isolation and characterization require careful attention under MISEV2023 recommendations [34]. These approaches are most useful when tied to a specific biological question, such as tissue injury or intercellular signaling. Their inclusion in a prediction model should follow evidence that they add useful information to more established measurements.

## 3. Human Evidence Across Cardiometabolic Applications

The human evidence does not form a single progression from discovery to clinical benefit. It falls into three distinct questions: whether measurements reflect relevant biology, whether they improve prediction beyond a clinical comparator, and whether acting on them improves outcomes. Most molecular–digital studies address the first question, some address the second, and very few address the third. The sections below therefore compare studies by the claim they can support rather than treating every association, response model or monitoring trial as evidence for an integrated dynamic-prediction system.

### 3.1. Glycemic Responses and the Development of Type-2 Diabetes

Glycemic regulation offers the clearest opportunity to combine molecular state with a continuously measured physiological response. The first requirement is to show that the two types of data provide complementary biological information. In 800 participants, postprandial glucose responses varied markedly between individuals eating the same foods, and personal and microbiome characteristics explained part of that variation [35]. PREDICT extended this observation in 1002 UK participants, with independent validation in 100 US participants, by relating glucose, insulin and triglyceride responses to meal composition, context and individual characteristics [36]. These studies establish that an apparently similar exposure can produce different metabolic responses and that molecular and personal information can help explain those differences.

Among 55 participants completing replicated challenges with seven carbohydrate meals, CGM responses varied with insulin sensitivity, beta-cell function, metabolites and proteins. The effects of fiber, protein or fat before a rice meal also differed by metabolic phenotype [37]. In 1398 participants, greater cumulative multimorbidity over 12 years was associated with current glycemic variability and response heterogeneity [38]. Even short environmental perturbations can alter the same system. In a randomized crossover study of 13 people with T2D, 4.5 days of natural daylight exposure during office hours, compared with artificial lighting, increased time within the study-defined glucose range of 4.4–7.2 mmol/L and shifted whole-body substrate use toward fat oxidation, although mean interstitial glucose did not differ [39]. Beyond these metabolic responses, multi-omic analyses identified daylight-associated differences in serum metabolites and lipids and in monocyte gene expression, while primary myotubes derived from skeletal muscle biopsies showed an advance in circadian phase. These findings illustrate how a short environmental perturbation can be accompanied by coordinated metabolic, molecular, and circadian responses. Together, these studies show how molecular state, prior health and current exposure shape short-term glucose responses. They do not yet show that combining these measurements predicts progression to T2D.

The second requirement is incremental prediction of future disease. A 44-metabolite signature added information beyond conventional risk factors across several cohorts, but the gain was smaller and less consistent after fasting glucose was included [40]. This result defines the appropriate test for integration. A combined molecular–digital model should be compared with a strong glycemic baseline that already includes fasting glucose, glycated hemoglobin or established CGM summaries. Otherwise, an apparent improvement may simply reflect information already available in routine care.

The third requirement is evidence that using the combined information improves care. MOBILE randomized 175 adults with T2D treated with basal insulin to CGM or blood glucose meter monitoring. At eight months, the adjusted between-group difference in glycated hemoglobin favored CGM by 0.4 percentage points, and severe hypoglycemia occurred in one participant in each group [41]. An 18-week personalized-nutrition trial in 347 participants improved selected measures, including triglycerides but not low-density lipoprotein cholesterol, through a package of personalization and behavioral support [42]. A meta-analysis of seven remote-monitoring studies involving 883 participants also found improved glycemic control, although the technologies, feedback and usual care differed [43]. These studies show that digital monitoring can be beneficial when it is linked to an effective response. They leave the central integration question open: whether molecular information improves the prediction or the resulting intervention beyond established digital and clinical data.

### 3.2. Atherosclerotic Cardiovascular Disease

Large cohort studies indicate that molecular data can improve cardiovascular prediction, although the gain varies by outcome and by the clinical information already in the model. In 41,931 UK Biobank participants, models based on 2923 proteins improved prediction over basic clinical models for 67 of 218 diseases and outperformed models that also included clinical blood assays for 52 diseases. External evaluation in EPIC-Norfolk covered six diseases [44]. In 50,057 participants, the area under the receiver operating characteristic curve was 0.767 for a proteomics-only model and 0.785 after clinical features were added [45]. Among 297,463 participants, adding clinical biomarkers, metabolomic scores and polygenic scores to SCORE2 increased the C-index by 0.014, 0.010 and 0.009, respectively. Combining all three produced an increase of 0.024 (95% confidence interval 0.022–0.027), with routine clinical biomarkers making the largest individual contribution [46]. In a reserved UK Biobank multi-omics sample of 24,287 participants, CardiOmicScore likewise found that proteomic scores generally discriminated better than metabolomic scores across six cardiovascular outcomes, while the incremental contribution depended on the endpoint and comparator [47]. These findings support endpoint-specific evaluation and explicit reporting of what each molecular layer adds to established clinical prediction.

Repeated molecular assessment asks whether change in a score can guide care. A 27-protein model for four-year risk of myocardial infarction, stroke, HF or death was independently validated in 11,609 participants, and estimated risk changed in the expected direction with weight loss, exenatide and disease-related changes [48]. This responsiveness is encouraging but does not establish the score as a surrogate endpoint. Treatment-induced changes in a valid surrogate should track treatment effects on clinical outcomes across relevant interventions. Clinical utility would then require a trial in which the score informs treatment.

Digital epidemiology adds exposure and functional context, but most available evidence remains between-person and observational. In UK Biobank, both a weekend-warrior pattern and a more evenly distributed active pattern were associated with lower risks of atrial fibrillation, myocardial infarction, HF and stroke than inactivity, suggesting that total activity volume may matter more than its weekly distribution [49]. In All of Us, higher step counts among 6042 participants with a median of four years of Fitbit monitoring were associated with lower risks of several chronic conditions [50]. These cohorts extend observation beyond a single clinic visit, but device ownership, engagement and underlying health shape both participation and measured activity.

Proteomic signatures associated with accelerometer-measured sedentary behavior or physical activity link the molecular and digital strands and were themselves associated with subsequent disease risk in UK Biobank [51,52]. The interpretation is limited when blood collection and accelerometry occur at different visits: the two modalities may characterize the same person without characterizing the same physiological state. Repeated, temporally aligned measurements are therefore needed before these signatures can be used to study within-person mediation or imminent events.

### 3.3. Heart Failure and Short-Term Deterioration

HF poses a different prediction problem from long-term primary prevention because clinically important changes may unfold over days. LINK-HF followed 100 patients after HF hospitalization with a multisensor patch and reported sensitivity of 76–88% at 85% specificity for detecting precursors of hospitalization, with a median warning interval of 6.5 days [53]. That interval could permit assessment before admission, but the study was small, predominantly male and based on wearable monitoring alone. It establishes feasibility of an early-warning signal, not the benefit of responding to it or of adding multi-omics.

Molecular measurements might make such warnings more interpretable. Natriuretic peptides, markers of injury and renal function already contribute to HF assessment, while broader proteomic and metabolomic profiles may capture myocardial stress, inflammation, substrate use and systemic dysfunction [1,9]. For example, falling activity with a rising congestion marker may prompt a different assessment from falling activity during an uncomplicated musculoskeletal illness. The relevant comparison is a broad panel versus a small set of established tests, with targeted sampling at an indicated encounter as a practical alternative to frequent venipuncture.

TIM-HF2 shows what is required to move from warning to benefit. Among 1571 randomized patients, structured remote management reduced the percentage of days lost to unplanned cardiovascular hospitalization or all-cause death relative to usual care [54]. Daily measurements of weight, blood pressure, electrocardiography and self-rated health were linked to organized clinical management through a continuously available telemedical service. Molecular–digital alerts should be evaluated in the same way, together with staffing, communication, confirmatory assessment and treatment response.

### 3.4. What Longitudinal Integration Has Established

Longitudinal integration has so far established that modalities contain related but nonredundant information. It has not established that combining them improves event-based decisions. In 96 participants followed for 16 months, five study visits, multiple molecular layers and continuous activity and sleep monitoring identified distinct sources of molecular variation and relationships with physiological and behavioral measures [55]. This is valuable longitudinal phenotyping and it supports retaining modality-specific information in later models, but event prediction and omics-guided care were not tested.

Longitudinal use also requires a distinction between differences among people and changes within the same person. Protein associations with physical activity and ethnicity identified in UK Biobank were examined over 48 months in a selected 58-person subgroup of the PROPELS trial. Interleukin-6 was the only activity-associated protein reproduced, whereas several ethnicity-related associations were retained [56]. Although the selected sample was small, the result illustrates that a marker separating active from inactive people may not track an individual’s response when activity changes.

This distinction helps define a practical role for wearable data. Wearable signals can estimate aspects of contemporaneous laboratory state [57] and may identify periods when a blood sample would be most informative. The resulting assay could then distinguish competing explanations for the digital change. Whether such coordinated sampling improves efficiency, prediction or outcomes remains to be tested. Table 2 organizes representative studies by their task, timing and principal limitation.

## 4. From Repeated Measurements to Dynamic Prediction

### 4.1. Defining the Prediction Task and Aligning Measurements

For a first target event, let T denote event time, t the current prediction landmark and h the prespecified prediction horizon. A dynamic model estimates r(t,h) = P(t < T ≤ t + h | T > t, H(t)), where H(t) contains the information available by t. At the next landmark, the history and at-risk status change and the model produces a new estimate [10]. The horizon can remain fixed, such as risk over the next 30 days, while the calendar window moves forward. Updating a patient’s prediction does not require refitting the model coefficients. Recurrent events or transitions between disease states require their own risk-set definitions.

In the proposed framework, H(t) combines relatively stable genomic susceptibility with current clinical factors, treatment history and time-indexed molecular and digital features. Genomic information can usually be retained across updates. A targeted protein or metabolite panel contributes its most recent available value and, when samples have been repeated, its change over time. Wearable and CGM records contribute prespecified summaries from a recent window, including deviations from a personal baseline. These inputs may enter a common outcome model directly or through the representations described in Section 4.2.

Temporal alignment depends on when a measurement was collected and when its result became available. At each landmark, the model should use only results available by that time. An intermittent assay may remain informative at later landmarks, but the model should retain its sampling date and evaluate how quickly its relevance declines. Digital features should be calculated from windows that end at or before the landmark and meet prespecified wear-time and quality criteria. Meal-related CGM features and molecular challenge responses should also be aligned to the same exposure. Measurements collected years apart may characterize the same person, but they should not be treated as a concurrent physiological profile.

For example, a study in stable HF could update 30-day hospitalization risk each week using clinical history, the latest available protein or metabolite panel and wearable summaries from the preceding seven days. A laboratory result reported after a weekly landmark would first enter the following update. Between molecular samples, the model would retain the age of the last assay while updating the digital history. These intervals illustrate the design logic rather than a validated schedule.

Retrospective analyses must reproduce the information available at each decision time. Interpolation, smoothing or feature construction that uses a later sample can leak future information into an earlier prediction. Preprocessing, imputation and feature selection should be learned within development folds, and repeated records from one participant should stay in the same fold [58,59]. Models should be evaluated when a modality is missing or an assay is old, including any rule to withhold an update. The observation process also needs attention when worsening health prompts extra sampling, as discussed in Section 4.3.

### 4.2. Selecting a Model That Fits the Data

Fusion can occur at the feature, representation or prediction level [60]. The event count, overlap between modalities and frequency of measurement should guide the choice. Table 3 compares practical considerations for longitudinal cardiometabolic datasets.

The simplest credible model should serve as the benchmark. When blood samples are infrequent and wearable records are dense, this may be a landmark model containing a targeted molecular panel and recent wearable summaries. More complex fusion methods should improve on that benchmark under the missing-data patterns and update schedules expected in practice.

Representation methods are useful when the feature space is too large for direct inclusion. MOFA+ summarizes shared and modality-specific variation across molecular data views [61], while MEFISTO extends this framework to temporal or spatial dependencies [62]. These methods organize measurement structure but do not by themselves predict clinical events. Their factors must enter a separate outcome model, and all preprocessing and factor estimation should be learned in development data. Any temporal interpolation used for prediction must exclude observations collected after the relevant landmark.

The outcome and sampling process then determine the longitudinal model. Landmark models estimate risk at successive prespecified times using information available at each landmark. Joint models link a longitudinal biomarker process with a time-to-event outcome, often through the estimated current level or slope [10]. They can account for measurement error in the estimated trajectory, but they do not automatically solve informative visit timing or missing modalities. Flexible sequence models may be useful in larger datasets, but they should be compared directly with simpler approaches. Sample-size planning should reflect the number of independent participants and events, candidate parameters, within-person correlation, censoring and the update schedule [63].

### 4.3. Missing Data, Treatment and Model Interpretation

Missingness carries clinical and behavioral meaning. A participant may stop wearing a device because of skin irritation, hospitalization or reduced engagement, while blood tests may become more frequent when clinicians suspect deterioration. These mechanisms differ from random technical loss. Study reports should describe participation in each modality, changes in observation over time and the assumptions underlying imputation. Models that operate with a limited set of modalities may be practical when some measurements are unavailable, provided their performance is evaluated under the missing-data patterns expected in care [59,60].

Treatment history is essential because medication initiation, discontinuation, dose changes and adherence can alter molecular concentrations, digital trajectories and event risk at the same time. A post-treatment fall in a marker may represent a pharmacological response rather than reversal of the underlying disease process. Conversely, worsening measurements may prompt effective treatment and weaken the observed association with later events. Dynamic models should therefore record treatment class, dose and timing, and adherence where feasible. Analyses can include time since treatment change and assess performance separately during stable and changing-treatment periods. Longitudinal multi-omics studies have documented such treatment-related molecular and physiological changes [55].

Prediction under observed care is distinct from estimating what would happen under an alternative treatment. The latter requires explicit causal assumptions and methods that address time-varying confounding, particularly when earlier measurements affect treatment and treatment then affects later measurements and outcomes [64,65]. For a prediction model, the immediate requirement is narrower: preserve treatment timing, avoid interpreting treatment response as untreated disease progression, and evaluate the system prospectively once its alerts begin to change care.

Biological interpretation can guide feature selection and identify plausible mechanisms, but model explanations should be read in the context of correlated measurements. A protein with high predictive importance may represent renal clearance, inflammation or disease severity rather than a treatment target. Genetic evidence and causal-network analyses can help prioritize hypotheses, as illustrated by work linking protein networks to subsequent myocardial infarction [66]. Such evidence is most informative when it converges with longitudinal or experimental findings. Prediction and mechanism can support one another without requiring every useful predictor to be causal.

## 5. Clinical Validation and Implementation

### 5.1. Establishing the Added Value of Integration

The appropriate comparator is the information a clinician would otherwise use for the same patient and outcome. In primary prevention, this may be a validated cardiovascular risk equation supplemented by routinely available tests. For glycemic management, it may include glycated hemoglobin, treatment and existing CGM summaries. For HF deterioration, it should include symptoms, recent hospitalization, treatment, renal function and established biomarkers. For dynamic prediction, the clinical comparator should use the same decision times, available routine history and prediction horizon as the integrated model [3,45,59].

Prespecified comparisons should evaluate clinical information alone, clinical plus molecular information, clinical plus digital features, and the combined model in the same evaluation participants and risk sets. Removing one layer from the combined model tests its incremental contribution conditional on the others, although it does not by itself establish a biological interaction. Baseline-only information, the latest available values and the full measured history should also be compared at the same landmarks and prediction horizons. This separates the value of adding a modality from the value of adding repeated measurements. Variables and model settings should be selected without using the evaluation data, so that reported performance reflects new participants rather than choices optimized to the test sample.

Discrimination assesses the ranking of event risk. Studies should report a horizon-specific, censoring-aware AUC or an appropriate concordance measure, together with the difference between models and its confidence interval [10]. Calibration assesses agreement between estimated risk and observed event frequency. At relevant landmarks and horizons, studies should report a calibration plot, calibration-in-the-large and calibration slope, including clinically relevant risk ranges and subgroups [67]. A Brier score can summarize prediction error at the chosen horizon. Evaluation must account for censoring and, when relevant, competing events. Uncertainty estimates should also retain the clustering of repeated predictions within participants.

Decision-curve analysis evaluates net benefit across thresholds linked to actual actions, using the threshold to weight false-positive decisions relative to true positives [68]. Compare the integrated model with the clinical model and appropriate act-on-all and act-on-none strategies. For time-to-event outcomes, use methods that account for censoring and relevant competing events [69]. Estimated net benefit describes potential usefulness under the specified decision assumptions. Prospective studies must establish whether using the model improves patient outcomes, with costs and participant burden evaluated alongside those outcomes. A statistically detectable gain in AUC alone is insufficient.

For repeatedly updated systems, evaluation must reflect repeated opportunities to alert. Performance per recording or per day may not describe a patient’s experience over months. Useful measures include warning time, the proportion of important events detected, and the number and duration of false-alert episodes per patient. Event adjudication and alert-episode definitions should be prespecified. TRIPOD+AI guides transparent reporting of prediction-model development and evaluation using regression or machine learning [58]. PROBAST+AI supports appraisal of study quality, risk of bias and applicability [59]. Reports should make participant selection, measurement timing, handling of missing data and the full evaluation procedure available for scrutiny.

### 5.2. Transportability and Representation

Independent evaluation is particularly important for molecular–digital models because both biology and measurement can vary across settings. Differences in age, ancestry, disease burden, treatment, assay platform and device use can change predictor distributions and baseline risk. A random split from one biobank is useful for internal evaluation, but it preserves many of these characteristics. Geographic and temporal validation, followed by prospective use in the intended setting, tests progressively more of the conditions the model will encounter. When recalibration is required, its performance should be evaluated separately from that of the original model [59,67].

Population representation also affects biological interpretation. Recent proteogenomic work in individuals from continental Africa expands evidence relevant to T2D beyond populations of predominantly European ancestry [70]. Digital cohorts face a related selection problem when participation depends on owning and consistently wearing a device. The 2026 All of Us wearables resource included data from 59,018 participants, combining donated device data with a program that supplied devices to participants [71]. This resource broadens opportunities to study longitudinal digital phenotypes and to examine how recruitment and device provision shape who is represented. Similar attention is needed when molecular sampling is added, because the cost and burden of repeated testing may create a second selection step.

### 5.3. Connecting Predictions to Care

Implementation can be evaluated only when the action linked to a prediction is specified. A risk estimate may trigger confirmatory testing, planned review, preventive treatment or more intensive monitoring, and each action has a different balance of benefit, burden and harm. If the prediction is generated by a machine-learning or other AI-based decision-support system, DECIDE-AI provides guidance for its early prospective evaluation in the intended care setting [72]. The relevant questions are whether the required data are available, clinicians can interpret and act on the output, the system fits the workflow and its use is safe. These questions should be addressed before a larger effectiveness trial.

The experience of CGM and structured HF telemanagement suggests that benefit depends on the interaction between measurement and care [41,54]. Even a small statistical gain may be useful if it identifies patients whose management changes appropriately. Its value will be lower when existing information already leads to the same treatment decision. Evaluation should therefore include downstream testing, medication changes, adherence, patient-reported burden and resource use, alongside clinical outcomes. Table 4 summarizes the questions connecting technical development with the intended decision.

Real-world implementation evidence comes from Xiao and colleagues’ retrospective study of 40,216 adults in a Chinese digital-health multidomain lifestyle program [73]. The program combined app-based monitoring and wearable data with professional support and offline wellness centers. Greater engagement was associated with larger improvements in blood pressure, blood glucose and body fat among relevant higher-risk subgroups. These findings illustrate digital monitoring within ongoing care, with self-selection, differential engagement and residual confounding affecting causal interpretation. The study informs implementation of a multidomain program, while the contribution of molecular–digital prediction requires separate evaluation.

Economic and participant costs should be evaluated alongside outcomes. Relevant resources include assays, sample collection and transport, devices and replacement, data infrastructure, clinician review and downstream investigations. Participant time, discomfort, skin reactions, travel and digital access can affect adherence and representation. Studies should compare targeted with broad panels and scheduled with clinically triggered sampling, report completion and dropout by population group, and estimate incremental costs against usual care. Cost per quality-adjusted life-year is appropriate when supported by outcome and utility data. Earlier feasibility studies can report observed costs and burden, clearly distinguishing projected long-term benefits from measured outcomes.

Operational reliability affects model performance after deployment. Systems need to communicate unavailable data, distinguish stable high risk from a new change and provide sufficient context for clinicians to interpret alerts. Assay updates and device firmware changes can alter features even when a patient’s biology is unchanged. Version tracking, calibration monitoring and a defined revalidation process help maintain clinical validity [20,58]. Data governance should support these activities while restricting access to identifiable molecular and behavioral information. Sustainable use also depends on a measurement schedule and workflow that patients and clinical teams can maintain.

## 6. Discussion and Future Directions

The evidence supports complementarity, but not yet routine integration. Large molecular cohorts identify signatures associated with long-term disease risk, and intensive studies show how molecular state varies with meals, activity, infection and other perturbations. Trials of CGM and structured telemanagement demonstrate that monitoring can improve outcomes when measurements trigger an effective clinical response. What remains uncertain is whether repeated molecular information improves that response beyond strong clinical care and established monitoring. The most defensible initial applications are therefore narrow ones in which the added modality addresses a recognized uncertainty, such as unexplained meal-response variation, residual risk after conventional assessment or changing functional status.

This evidence also argues for separating discovery from deployment. Broad multi-omics panels are useful for identifying pathways and candidate combinations. A clinical system may need only a targeted panel chosen for stability, accessibility and incremental information. Dense digital records need not be merely another predictor stream. They can identify when molecular sampling is most informative, while the assay can distinguish competing explanations for the digital change. Biomolecular profiling and wearable sensing now make this coordinated design technically plausible [7], but its efficiency and clinical value must be measured rather than assumed.

Emerging technologies should be interpreted according to the problem they have solved. Active-reset protein sensors enable repeated in vivo monitoring of inflammatory proteins in animals, and an epidermal platform can track cholesterol and triglycerides in sweat while experimentally examining their relationship with blood lipids [74,75]. These are advances in sensing feasibility. Before they become cardiometabolic predictors, studies must establish local-to-systemic relationships, calibration during ordinary use, stability, wear burden and incremental information beyond established blood tests. A transient signal matters clinically only if it improves prediction or action relative to simpler periodic sampling.

Dynamical network biomarkers and digital twins are further from clinical evaluation because their claims concern system transitions and counterfactual response. A dynamical network biomarker combines increasing variability and within-network correlation with decreasing correlation to the surrounding network under specified conditions [76]. In cardiometabolic disease, infection, treatment, sampling artifacts and reversible physiological responses can produce similar changes. Prospective studies should therefore define the transition, compare the network signal with simpler trends and validate it in new participants. Digital twins combine mechanistic models with patient-specific observations to simulate a defined cardiovascular process or treatment scenario [77]. Molecular and wearable measurements could inform their parameters, but validation must address the particular simulated task, the uncertainty of the response and, for treatment decisions, outcomes under alternative actions [12,65]. Neither label should substitute for a testable prediction target.

The next studies should be organized around evidence gaps rather than technology categories. First, prospective cohorts should align molecular and digital observations around defined clinical transitions, with background and perturbation sampling to identify useful timing and personal baselines. Second, nested comparisons should test whether broad discovery panels can be reduced to targeted assays and whether repeat sampling adds value at an acceptable cost. Third, prospective decision studies, randomized where feasible, should compare the complete integrated care pathway with usual care, recording alert review, treatment changes, participant burden and resource use. Common outcomes, explicit sampling and treatment timelines would make these studies comparable.

This review is narrative and synthesizes heterogeneous populations, measurements and clinical objectives. It does not provide pooled estimates or a systematic risk-of-bias assessment. Observational evidence predominates, external validation is limited for some models, and few trials isolate the incremental value of integration. Those limitations preclude a claim that comprehensive multi-omics and continuous monitoring are ready for routine care. A more plausible near-term system is smaller: a strong clinical model, a targeted molecular panel and a limited set of repeatable digital features, tested in the patients and workflow for which it is intended.

## 7. Conclusions

Multi-omics and digital measurements answer different questions. Molecular assays characterize susceptibility and biological state, whereas digital trajectories show when and how physiology changes in daily life. Current human studies support these complementary roles and, in selected settings, improved prediction. They do not yet establish that repeated molecular–digital integration improves clinical outcomes. A credible dynamic system must specify its outcome, horizon, update time and clinical action. It must use only information available at each update and show what each modality adds beyond a strong clinical comparator. The field should now move from demonstrating that more data can be combined to testing whether a smaller, temporally aligned set of measurements changes decisions, improves outcomes and justifies its cost and burden.

## Figures and Tables

**Table 1:** Information contributed by molecular and digital measurements to cardiometabolic assessment.

Measurement	Main Information	Longitudinal Use	Interpretation
Genomics	Inherited susceptibility; context for molecular associations	Usually assessed once; combined with changing clinical state	Predictive value varies with ancestry and comparator. Ancestry-specific evaluation and, when needed, recalibration are important.
Proteomics	Signaling, inflammation, organ stress and remodeling	Repeated panels or selected proteins around changes in health	Abundance reflects production, release and clearance; platforms may differ.
Metabolomics and lipidomics	Substrate handling and integrated metabolic state	Fasting profiles, meal challenges and intervention responses	Diet, sampling time, medication and organ function affect concentrations.
Other molecular layers	Transcriptional regulation, epigenetic state and host–microbe relationships	Mechanistic characterization and selected longitudinal applications	Cell composition, specimen source and preanalytical handling are important.
Wearable physiological signals	Activity, rest-activity rhythms, heart rate and rhythm	Repeated summaries, trends and responses during daily life	Context, wear time, device version and measurement method affect features. Biomarker use requires evidence for the specified biological indication.
Continuous glucose monitoring	Interstitial glucose excursions, variability and recovery	Meal responses, daily patterns and treatment monitoring	Specify whether glucose is a predictor, response or endpoint. Validate the derived metric for its intended population and use.

Measurement schedules should follow the clinical question and the biological process. The predictive contribution of each measurement class requires evaluation in the combined model. Relevant references are [4,5,13,17,18,19,20,21].

**Table 2:** Representative human studies informing molecular–digital cardiometabolic assessment.

Study and Population	Data and Timing	Clinical or Biological Task	Main Contribution	Principal Limitation for Integrated Dynamic Prediction
Marabita et al.; 96 participants [55]	Repeated multi-omics and digital monitoring over 16 months	Longitudinal phenotyping	Identified distinct molecular and physiological dimensions	Small phenotyping cohort. External event-prediction validation and trials of omics-guided care remain needed.
Wu et al.; 55 participants [37]	CGM, replicated meal challenges, metabolic tests and multi-omics	Meal-response heterogeneity	Linked responses to metabolic physiology	Small mechanistic study with meal-response endpoints. Prospective diabetes-risk validation remains needed.
Daylight crossover trial; 13 participants with T2D [39]	CGM and molecular profiling during two 4.5-day conditions	Short-term environmental response	Linked daylight exposure to glucose-range and substrate-use changes	Very small, short trial focused on metabolic responses. Long-term outcomes and predictive performance remain uncertain.
Li et al.; multicohort study [40]	Baseline metabolomics with follow-up	Incident T2D prediction	Evaluated a 44-metabolite signature across datasets	Incremental value depended on adjustment for fasting glucose. Repeated wearable updating and score-guided care were outside the study scope.
Carrasco-Zanini et al.; 41,931 UK Biobank participants [44]	Baseline proteomics and clinical information	Multiple-disease prediction	External evaluation of selected disease models	External evaluation covered six diseases. Repeated molecular and wearable updating requires further study.
CardiOmicScore; 24,287 in the reserved multi-omics sample [47]	Baseline molecular, genetic and clinical predictors	Incident cardiovascular outcomes	Compared endpoint-specific combinations	Reserved and geographic testing used UK Biobank. Independent-population validation and repeated wearable updating remain needed.
Henson et al.; UK Biobank and 58 PROPELS participants [56]	Proteomics and activity change over 48 months in a selected subgroup	Within-person activity-related biology	Reproduced the IL-6 activity association	Small selected subgroup. Replication of activity-related proteins was limited to IL-6. Event prediction was outside the study scope.
LINK-HF; 100 patients [53]	Continuous wearable monitoring after HF hospitalization	Short-term deterioration	Reported a clinically relevant warning interval	Small, predominantly male cohort. Wearable-only design with observational evaluation of alerts.
MOBILE; 175 adults using basal insulin [41]	Randomized CGM versus meter monitoring over eight months	Glycemic management	Improved HbA1c with a monitoring strategy	Specific treatment population and glycemic endpoint. Molecular contributions and cardiovascular outcomes were outside the trial scope.
TIM-HF2; 1571 randomized patients [54]	Structured remote monitoring and clinical management	HF care outcomes	Tested a complete monitoring-and-response program	Evaluated a complete care program. The effects of individual sensors and multi-omics integration require separate comparisons.

Studies are grouped by the contribution they make to integrated assessment. Molecular-only and digital-only studies provide evidence for individual components or care pathways, and their different endpoints preclude direct performance ranking. Abbreviations are CGM (continuous glucose monitoring), HF (heart failure), T2D (type 2 diabetes), HbA1c (glycated hemoglobin) and IL-6 (interleukin-6).

**Table 3:** Practical comparison of fusion strategies for longitudinal cardiometabolic data.

Strategy	Potential Advantage	Main Constraint	Practical Fit
Early integration	A penalized model can combine a small set of clinical, molecular and digital features with selected interactions.	Requires aligned observation windows. High dimensionality increases overfitting, and sparse modalities complicate imputation.	A targeted panel and prespecified digital summaries with adequate overlap provide a benchmark when events are limited.
Intermediate integration	Shared and modality-specific representations summarize high-dimensional data. Temporal methods accommodate longitudinal structure.	Small cohorts can produce unstable representations. Batch and treatment effects may be retained while weak outcome signals are lost.	Repeated datasets with sufficient participants and events, explicit temporal structure and separate development and evaluation samples.
Late integration	Separately developed modality-specific models can be combined and updated at different frequencies.	Cross-modal interactions may be missed. Risk estimates require a common horizon, calibration and a specified approach to missing modalities.	Incomplete overlap or asynchronous sampling, with validated component models and evaluation of each available-modality pattern.

The strategies are not mutually exclusive, and a study may combine them. The preferred approach depends on the prediction task, event count, temporal alignment and overlap between modalities.

**Table 4:** Evaluation questions for a dynamic molecular–digital prediction system.

Question	Design or Comparison	Decision-Relevant Output
Does each modality add information?	Compare clinical-only, clinical-plus-molecular, clinical-plus-digital, and all three in the same participants, risk sets and horizons.	Changes in discrimination and prediction error with uncertainty; calibration and net benefit of the combined model.
Does repeated measurement help?	Compare baseline information, latest values and measured histories at matched landmarks and horizons.	Value of trajectories, sampling intervals and update frequency.
Are predictions temporally valid?	Use only results available at each prediction time, including assay turnaround. Keep each participant within one development or evaluation split.	Performance at the intended horizon without future-information leakage.
Would decisions improve?	Assess horizon-specific calibration and net benefit against the clinical model and act-on-all/none strategies at actionable thresholds.	Expected balance of useful actions, missed events and unnecessary follow-up.
Does the system transfer?	Evaluate new populations, times, devices and assay settings; assess incomplete-data operation.	Transportability, subgroup performance and recalibration needs.
Does the care pathway work?	Prospectively test the model together with the clinical response and workflow.	Patient outcomes, alert burden, resource use and sustained adoption.
Is the gain worth the cost and participant burden?	Compare targeted and broad panels, and alternative sampling schedules, against usual care. Include assays, devices, clinical review, downstream testing, participant time, discomfort, adherence and dropout.	Incremental cost and, where supported, cost per quality-adjusted life-year. Sampling completion, patient-reported burden and distribution of costs and benefits across groups.

Together, these questions adapt established prediction-model and clinical-evaluation guidance to integrated cardiometabolic monitoring [58,59,67,68,72].

## Data Availability

No new data were created or analyzed in this study. Data sharing is not applicable to this article.
